# The dynamics of audience applause

**DOI:** 10.1098/rsif.2013.0466

**Published:** 2013-08-06

**Authors:** Richard P. Mann, Jolyon Faria, David J. T. Sumpter, Jens Krause

**Affiliations:** 1 Department of Mathematics, Uppsala University, Uppsala 75106, Sweden; 2 Institute for Futures Studies, Stockholm 11136, Sweden; 3 Institute of Integrative and Comparative Biology, University of Leeds, Leeds LS2 9JT, UK; 4 Department of the Biology and Ecology of Fishes, Leibniz Institute of Freshwater Ecology and Inland Fisheries, 12587 Berlin, Germany

**Keywords:** social contagion, SIR model, applause, clapping, Bayesian model selection

## Abstract

The study of social identity and crowd psychology looks at how and why individual people change their behaviour in response to others. Within a group, a new behaviour can emerge first in a few individuals before it spreads rapidly to all other members. A number of mathematical models have been hypothesized to describe these social contagion phenomena, but these models remain largely untested against empirical data. We used Bayesian model selection to test between various hypotheses about the spread of a simple social behaviour, applause after an academic presentation. Individuals' probability of starting clapping increased in proportion to the number of other audience members already ‘infected’ by this social contagion, regardless of their spatial proximity. The cessation of applause is similarly socially mediated, but is to a lesser degree controlled by the reluctance of individuals to clap too many times. We also found consistent differences between individuals in their willingness to start and stop clapping. The social contagion model arising from our analysis predicts that the time the audience spends clapping can vary considerably, even in the absence of any differences in the quality of the presentations they have heard.

## 1. Introduction

Mathematical models of social contagion have been suggested for everything from pop songs and fashion to divorce and suicide [1–3]. Each social contagion model has its own set of assumptions about how individuals are ‘infected’ by others [4]. In general, these assumptions have not been tested experimentally, leaving several key empirical questions unanswered about how humans respond to each other [5]. For example, does the probability of social infection increase in proportion to the number already infected, as it does in most models of disease epidemics? Or is there a tipping point at which infection takes off? Do fashions die out because they have been around for too long or is there a socially mediated ‘recovery’? Are local neighbours or the proportion of the total population who are infected most important in spreading ideas?

Recent work has begun to quantify social contagion in, for example, joining of social networks [6] and gaze following [7]. However, human social dynamics remain notoriously difficult to quantify [8–10] and new methods are required to identify which cues people are responding to. One natural group setting, where it is relatively easy to quantify collective behaviour of humans is in audience applause, where previous studies have empirically investigated the emergence of self-organized rhythmical patterns [11,12]. Here, we quantify the role of social contagion in the start and stopping of applause. In an applause setting, each clap produced by an individual provides us with a time point at which he or she remains ‘infected’ by appreciation, and cessation of clapping denotes ‘recovery’. This type of datum allows us to apply a Bayesian model selection approach to determine the dynamics of how social cues spread through group members. Although, as with any other statistical method, we cannot conclusively rule out the influence of unobserved confounding variables, our approach allows us to accurately select which of the observed cues are the most probable cause of the social contagion and avoid identifying spurious but statistically significant correlations to confounding variables when multiple observed cues are correlated with each other.

## 2. Results

**Figure 1.**
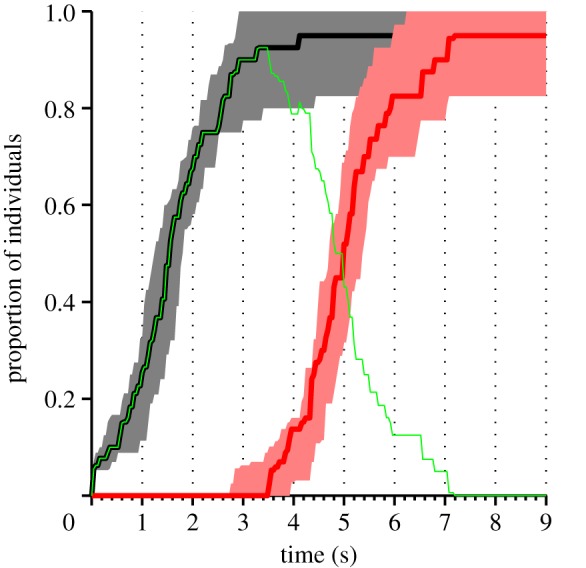
Experimental results. The plot shows the median proportion of individuals in the audience who have started clapping (black line), stopped clapping (red line) and are currently clapping (green line), aggregated over the 12 experimental presentations. For the starting and stopping proportions, the shaded area represents the interquartile range, illustrating the variation across experiments.

We filmed the response of groups of 13–20 university students to an oral presentation. Six different groups (consisting of a total of 107 students) listened to two presentations each (see §4 for details). A group's clapping can be ordered in terms of starting clapping (figure 1, black line) and stopping clapping (figure 1, red line). After the presentation was completed, the mean duration for the first person to begin clapping was 2.1 s (±s.e.: 0.62 s). The mean interval from the first person to start clapping, to the last person to start was 2.93 s (±s.e.: 0.33 s). The mean applause length (from the first person to start clapping to the last person to stop) was 6.1 s (±s.e.: 0.27 s). The mean duration for the first person to stop clapping was 5.56 s (±s.e.: 0.74 s), and the mean duration from the first person to stop clapping, to the last person to stop was 2.6 s (±s.e.: 0.3 s).

**Figure 2.**
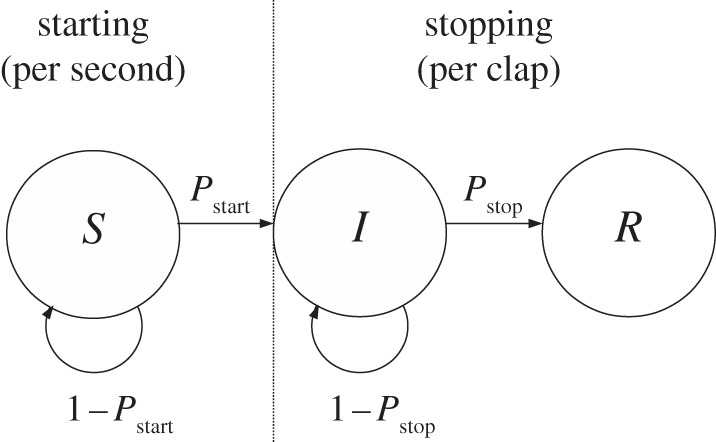
Model schematic. Individuals progress from an initially ‘susceptible’ state (*S*), before they have started clapping, to an ‘infected’ state (*I*) while clapping and eventually to a ‘recovered’ state (*R*) once they stop clapping. The probability of moving from *S* to *I* is given by the starting probability per second, *P*_start_. Once the individuals have started clapping, they either stop or continue after each successive clap, stopping with probability *P*_stop_ or continuing with probability 1 − *P*_start_. These probabilities are determined by the proportion of individuals and direct neighbours who have started and stopped clapping and the number of claps each individual has already performed according to the models described in the text.

Both the onset and the cessation of clapping follow a sigmoidal curve, with an initially slow uptake of the new behaviour followed by a phase of rapid change and eventual saturation (figure 1). Such sigmoidal growth and decay resemble the pattern of infection typically seen in the spread of diseases, both empirically and in epidemiology models, supporting the possibility of social contagion in clapping. We used a Bayesian methodology to test models for starting and stopping clapping [13–15]. We construct models that specify the probability that an individual will start or stop clapping (figure 2). These probabilities are conditioned on the state of the group (see the listed group characteristics below). By iterating over all observed events (starting/not starting; stopping/not stopping) and multiplying the probabilities of those events specified by a given model, we determine the likelihood of the data conditioned on any specific values of the model's adjustable parameters. Further summing over a range of possible parameter values by integration, we fairly assess the relative probabilities of the models (see §4).

For starting clapping, we tested five alternative models. (*M*_1_) independent: the probability of starting clapping is a constant rate, independent of the clapping of others; (*M*_2_) linear response: the rate to start clapping depends on the proportion of audience, *ρ*_clapping_, who are already clapping; (*M*_3_) quadratic response: the rate to start clapping depends on 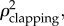 providing a threshold above which the probability of clapping significantly increases; (*M*_4_) nearest neighbours: the rate to start clapping increases when your immediate neighbours start clapping; (*M_G_*) first clap reaction: individuals wait for the first clap then start clapping with a normally distributed response time. The last four of these models are consistent with the sigmoidal increase in clapping seen in the data. We combined the models (1) to (4) into a single equation for the probability of starting, with adjustable parameters *λ*_*i*_, such that2.1
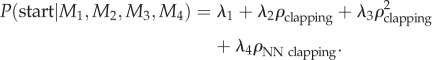
From this general equation, any combination of models (1) to (4) can be constructed by definitively setting a subset of the *λ* parameters to be zero, while allowing others to vary and be inferred from the data. For example, by setting *λ*_1_ = 0 and *λ*_3_ = 0, we specify a model where each individual can respond to the total proportion of clappers in the audience, and more strongly to those who are their nearest neighbours:2.2
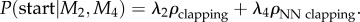
Thus, each potential model can be identified by specifying which elements remain active, in this example (*M*_2_) and (*M*_4_). Model (*M_G_*) specifies that the time to start clapping should be Normally distributed. The probability of starting during any specified time period is therefore given in terms of the cumulative Normal probability distribution, *Φ*(*x*, *μ*, *σ*), where *μ* and *σ* are the mean and standard deviation of the distribution of starting times.

The marginal likelihood was highest for the model involving a purely linear response (see the electronic supplementary material, figure S1*a*), i.e. model (*M*_2_). The rate at which new individuals begin clapping, after the first clap is made, is proportional to how many are already clapping, with an inferred value of *λ*_2_ = 2.15 ± 0.15 per second. The addition of any effect other than (*M*_2_) made the data less probable, indicating that these other cues were not involved in the decision to begin clapping. Further evidence for the importance of (*M*_2_) is demonstrated by the fact that all top six models include this term.

For the cessation of clapping we tested four models. (*M*_1_) independent (as above); (*M*_2_) linear response: the rate to stop clapping depends on the proportion of audience, *ρ*_stopped_, who have stopped clapping (not including those who have not started yet); (*M*_3_) increasing with clap number: the rate to stop clapping increases with the number of claps, *n*_claps_, already performed by the individual (in proportion to the maximum number of claps observed in experiments); (*M*_4_) nearest neighbours: the rate to stop clapping increases in proportion to the number of your immediate neighbours that stop clapping; (*M_G_*) preferred clap duration: individuals clap a normally distributed number of times independent of others in the group. Again, we also compared combinations of models *M*_1_ − *M*_4_, which can be combined to a single form:2.3
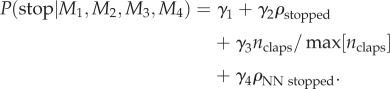
Similar to the case of the starting models, we can set any subset of the *γ* parameters to be zero to define a range of mixed cue models to test. Model (*M_G_*) has a form equivalent to the respective starting model, with mean number of claps *m* and standard deviation *s*.

The four models which fit the data best all involve a term *γ*_2_ > 0 for linear response, similar to the models for starting clapping. However, the best model combines (*M*_2_) with an increased cessation with clap number, i.e. *γ*_3_ > 0 (see the electronic supplementary material, figure S1*c*). The relative size of the parameters (the best fit *γ*_2_ = 0.63 is over 10 times as large as *γ*_3_ = 0.05) suggests that social contagion is a more important factor in stopping than the number of claps performed. Note that in this analysis a model with only (*M*_2_) is not possible, since at least one individual must stop clapping when no others have done so. Performing a similar analysis on a restricted subset of the data, after at least one audience member has stopped clapping, identifies a purely social model as the most probable (see the electronic supplementary material, figure S1*e*). This suggests that the non-social element relating to the number of claps performed serves to regulate the initiation of stopping, which is then mediated through a social process. It was not possible to identify precisely when each presentation was completed, so our analysis of starting behaviour begins after the first clap is performed.

**Figure 3.**
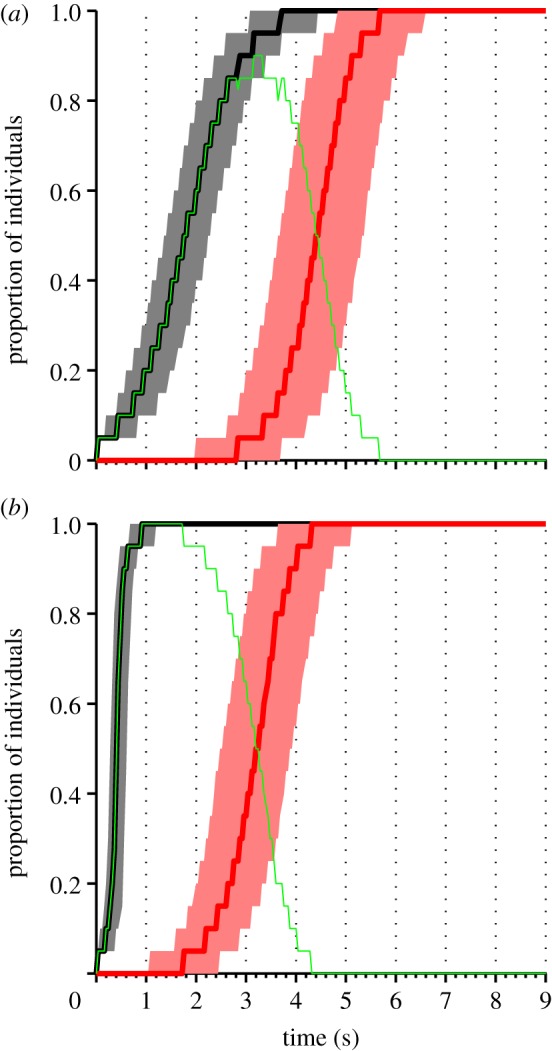
Simulation results. The plot shows the median proportion of individuals in the audience who have started clapping (black line), stopped clapping (red line) and are currently clapping (green line), aggregated over 10 000 simulations. Results are shown for (*a*) the optimal linear contagion model and (*b*) an alternative quadratic contagion model. For the starting and stopping proportions, the shaded area represents the interquartile range, illustrating the variation across simulations. The simulation has three behavioural states: susceptible (*S* is the proportion of individuals in this state); clapping (*I*) and recovered (*R*). The time taken to go from susceptible to clapping is exponentially distributed with rate constant *λ*_2_(*I* + *R*). After the *n*th clap an individual will either recover (stop clapping) with probability *γ*_2_*R* + *γ*_3_*n*/*n*_max_ or wait a time distributed *N*(0.28 s, 0.09 s) (matched to observed clap intervals). This process continues until all individuals have recovered.

Rules of interaction between individuals in groups that are inferred from fine scale measurements of individual behaviour should be confirmed by demonstrating their ability to reproduce group level effects [15–17]. To further investigate the dynamics of applause, we implemented a simulation model based on the combination of the most probable starting and most probable stopping models (see §4). This model reproduces the type of dynamics seen in the experiment (figure 3*a*). In particular, the model accurately reproduces the form of the sigmoidal starting and stopping patterns seen in the data and the approximately symmetric growth of decay of the infection. To test our hypothesis that clapping contagion is a linear process, we also performed simulations of a model with a quadratic infection term (*M*_3_ in the starting models), using the best-fit parameters for that model (*λ*_3_ = 4.0 per second). The results of these simulations (figure 3*b*) show that such a model is inconsistent with the large-scale pattern of infection seen in the data (figure 1). In particular, infection occurs too rapidly, and there is a sustained period where all individuals are clapping, whereas in the data and in the linear simulations some individuals typically stop clapping before the whole audience is infected.

**Figure 4.**
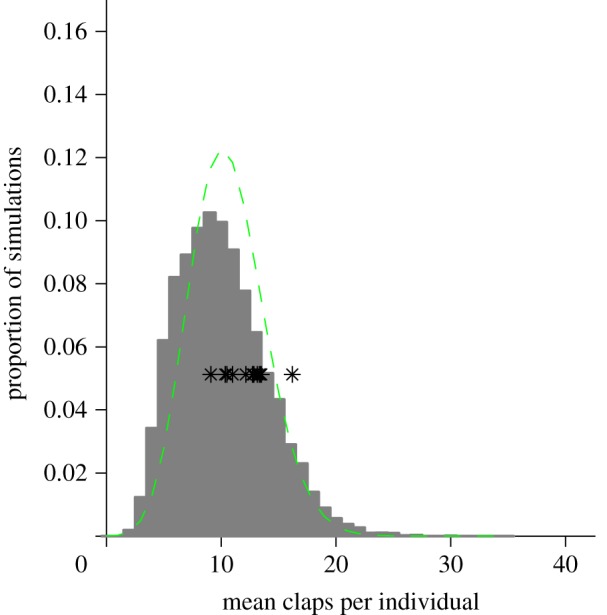
Distribution over 10 000 simulation runs of average number of claps performed per individual, shown with grey bars. All parameter values are the same as in figure 3*a*. The simulation is run 10 000 times and the average number of claps performed is recorded for each run. The figure is then a proportional distribution over all outcomes. Black stars indicate the mean number of claps performed per individual in each experimental presentation. The dashed line shows a Poisson distribution matched to the same mean as the simulated distribution, showing that shorter and longer bouts are more common in the simulation than expected under a Poisson model. (Online version in colour.)

An intriguing model prediction is that the length of time the audience spends clapping varies considerably (figure 4). Running the simulation multiple times, we see a large variability in the average number of claps across trials. While the majority of clapping bouts involve only 9–15 claps per person, some bouts can last over 30 claps. Compared with a Poisson distribution, the distribution arising from the simulation is more skewed towards both short and long bouts. This variability does not arise from any difference in the stimulus (i.e. the parameter values are the same for each simulation) but is rather a property of the social interaction involved in clapping and the variability in when stopping is initiated by through the non-social aspect of the model. The mean number of claps performed in each experimental presentation, indicated by black stars, all lie within the central 95 per cent of the simulated distribution.

We did not observe any difference in the type of response made to talk 1 and talk 2 (see the electronic supplementary material, figure S2 and the text therein for test details). We did, however, find a significant correlation between the order that particular individuals started clapping (*N*_individuals_ = 104; *N*_group_ = 6; median *ρ* = 0.53; *p* < 0.0001, randomized-ordering bootstrap on Spearman rank correlation coefficient) and stopped clapping (*N*_individuals_ = 104; *N*_group_ = 6; median *ρ* = 0.37; *p* < 0.0008) between the first and second presentations they listened to. This result indicates that the willingness to clap is characteristic of individuals.

## 3. Discussion

Unlike studies focused on visual information, where local transmission of information is between local neighbours [18–20], we find in our experiments that spatial proximity is not important. This is probably the result of attention to a less localized acoustic cue (i.e. the volume of clapping) instead of the behaviour of local neighbours. While the individuals were found to be increasingly likely to stop clapping as their clapping duration increased, we find that overall, global social influences appear to be more important than internal information in the decision to stop clapping. Because of the relatively weak but necessary effect of the individual duration of clapping upon probability of stopping, we suggest that this serves to regulate when stopping is initiated at the group level, with the stronger social effect subsequently mediating the rate of stopping after initiation. This interpretation is supported by the superior performance of a purely social model when considering the stopping process after the first person stops clapping.

**Figure 5.**
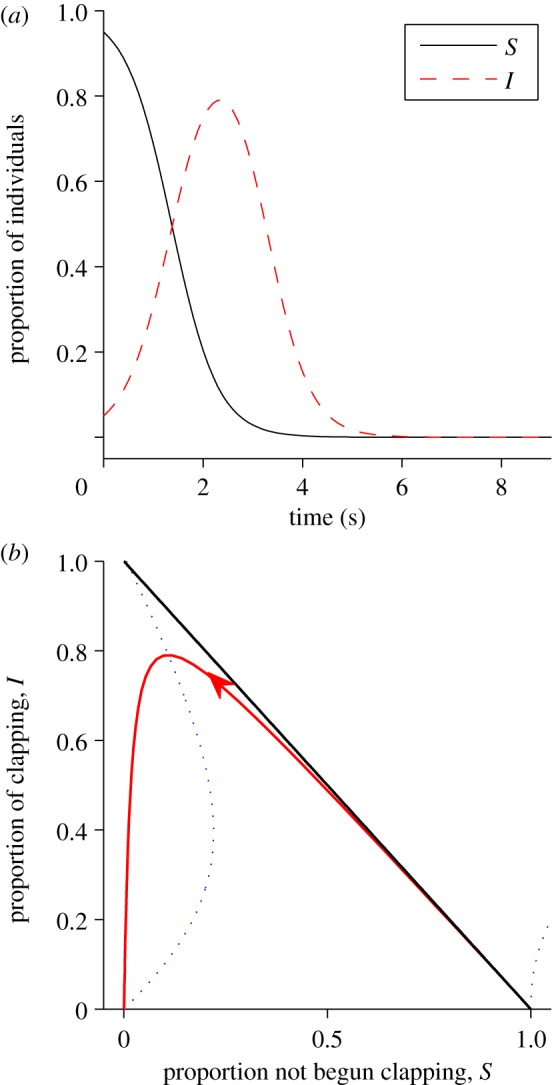
Ignoring the effect of number of claps on the probability of stopping, the clapping model can be expressed in terms of mean-field differential equations (4.4) and (4.5). The model parameters are set from the best model fit when ignoring clap number-dependent stopping (i.e. *γ*_3_ = 0), with stopping parameters adjusted from per clap values to per second values using the average clap interval of 0.28 s, yielding *γ*_2_ = 2.15 per second, *γ*_1_ = 0.0011 per second and *γ*_2_ = 0.094 per second. (*a*) Change of susceptible and infected individuals through time when *S*(0) = 0.95 and *I*(0) = 0.05. (*b*) Phase plane of susceptible versus infected. When 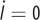 (indicated by the blue dotted line) clapping reaches a maximum. The red arrowed line shows the time integration from figure 5*a*.

While an analogy to disease spread is the starting point for describing social contagion, our study reveals important differences between biological and social processes. As in the standard Susceptible, Infected and Recovered (SIR) model for the spread of a disease [21], and in contrast to models based on tipping points or quorums [22,23], clapping increases linearly with the proportion of individuals already involved in it. This linear response is similar to that seen in movement decisions in monkeys [24] and in gaze-following by humans [7]. However, unlike the SIR model, ‘recovered’ individuals (those who have stopped clapping) increase the recovery rate of those who are clapping. This is consistent with an early model by Daley & Kendall [25] of fads and fashions. Figure 5 shows a phase plane for a differential equation version of our clapping model, in which the effect of number of claps on the probability of stopping is ignored. Unlike the SIR model, clapping always spreads even when the starting rate *λ*_2_ is small. The point at which most people are infected with clapping is near to the point at which there remain very few susceptibles, similar to both the experimental results (figure 1) and the stochastic simulations (figure 3). As seen in the experimental results (figure 1), the model predicts that even before everyone has started clapping, some individuals will usually have recovered and stopped.

Here, we have established a particular empirical form for social contagion in clapping. While we expect other social activities may have different functional forms, it is striking how well a simple model fits the data. The dynamic nature of clapping data and the model comparison method we have adopted here has allowed us to further determine the relative weights of internal cues (how long I have clapped) and external cues (how many others are still clapping) in the cessation of an activity. We believe that our methodology can be equally applied to other social contagion data. In psychological, economical and sociological phenomena, the confounding of many potential causes for given effect gives rise to statistical difficulties in inferring what proportion of the observed behaviour can be attributed to a given cue [26–29]. For example, the rate at which individuals leave social networks or online groups is likely to be a function of both how long a focal individual has been a member and the engagement of other members [2,30]. The methods presented here could be used to find the relative weighting of these internal and external cues and predict how long particular online fads will last. Under the Bayesian method, for any set of potential cues, the relative model likelihoods show which cue or combinations of cues best explain the data. Multiple cues must be sufficiently strong and independent to warrant their inclusion in the model [31, ch. 4 and 20].

Sociological phenomena that appear well understood on the level of the individual can be highly sensitive to stochasticity in individual responses, producing different patterns at the group level [32]. Similar phenomena are observed in collective animal behaviour, where fitting a model at the level of the individual does not immediately imply an understanding of global dynamics [15–17]. To address these potential problems, we tested the group level implications of the ‘rules of interaction’ we had inferred between individuals by simulation, and found that these gave rise to bouts of applause with a similar mean duration to the real talks, with a similar sigmoidal profile for both the initiation and cessation of clapping. Stochasticity in the individual responses led to variation in the total applause duration which mirrors the results of our experiments.

In our experiment, different talks did not cause differences in the length of clapping bouts. Our model suggests that variation in the length of applause can arise even for talks which are equally appreciated by the audience (figure 4). Randomness in the audience interactions can sometimes result in unusually strong or weak levels of appreciation, independent of the quality of the presentation. Groups must coordinate the cessation of clapping and on different occasions this can take longer or shorter periods of time to achieve. The social problem an audience must solve after an academic presentation is not how and when to start a round of applause, but it is rather how to coordinate its end.

## 4. Methods and material

### 4.1. Experiment

Experiments took place at the University of Leeds during March 2009. We observed the behaviour of participants in an audience in response to an oral presentation. We used 107 participants. The participants were university students and prior to the experiment we requested permission to video their behaviour. We also used six presenters who were undergraduate (but not in the same year as the audience participants) or postgraduate students. Participants and presenters were naive as to the purpose of the investigation.

Experiments took place in a small seminar room (10 × 12 m). A presenter stood at one end of the room in front of a large screen, and the audience was instructed to sit, facing the presenter, in three rows.

Participants were organized into six groups (three groups comprised 20 individuals, and a further three groups comprised 18, 16 and 13 individuals). Each group was assigned (at random) to attend two presentations by different presenters. The participants were told to observe and record the body language of the presenter, so that their attention was on the presenter and therefore they were less likely to consider that their clapping behaviour was under investigation. They were also told that they should applaud the presenter at the end of the presentation because the presenters were performing the presentation voluntarily.

For each of 12 presentations (two by each presenter), the presenter performed a 7 min oral Powerpoint presentation on a biological study. The end of the presentation was defined as when the speaker completed their final statement, such as ‘thank you’ or ‘any questions?’.

We observed the time at which participants in the audience started and stopped clapping from video footage. Clapping was defined as when an individual struck a part of their body with one of their hands in a repetitive manner (this was usually their other hand but in some cases it was their arm or their shoulder). An individual was considered to have started to clap at the point where their hands met for the first time during a clapping bout, and was considered to have stopped clapping when their hands met for the final time.

One hundred and four out of the 107 participants started to clap after the presentation.

### 4.2. Model comparison

We take a Bayesian model comparison approach to identifying the causes of both starting and stopping clapping, following the methodology of Mann *et al*. [15]. This means that we propose various hypotheses about how clapping starts and stops in the form of models. These specify the probability of an individual either starting and stopping clapping, as a function of the potential cues of interest such as the number of other clappers, number of clappers who have stopped and the length of time the individual has been clapping. Since the effect of those cues is unknown, the influence of each upon the starting or stopping probability is modulated by a set of free parameters, *ϕ*. By multiplying over the sets of all events 𝒳 (either time steps or claps), all individuals ℐ, and all experiments ℰ, each of these hypotheses, or models, *M_i_* thus specifies the probability of the complete data, *D*, conditional on the model and the parameter values,4.1
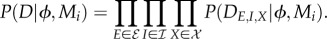
Different hypotheses use a different set and number of terms to represent different cues, and the sensitivity to the exact parameter values varies between models. To account for this, we integrate over the unknown parameters, using a reasonable (see below) prior probability distribution, *P*(*ϕ*|M_*i*_), to find the probability of the data conditioned only on the model.4.2
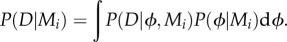
By a ‘reasonable’ prior distribution, we mean one that has significant probability mass across a broad range of possible parameter values and which does not either specify strong knowledge about any specific parameter value or favour one model over another by design. See the electronic supplementary material for details of the precise prior distributions used for each parameter.

We select among possible hypotheses by asking which is most probable in the light of the data. Assuming all models to be equally probable *a priori* this means that we select the model with the greatest marginal likelihood, argmax*_i_P*(*D|M_i_*). To evaluate the integral in equation (4.2), we use importance sampling Monte Carlo (see standard texts [33]). We draw *N* random samples, 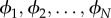, from the prior distribution of the parameters. The integral is then approximated as4.3
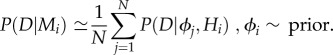
The uncertainty in this estimate scales as 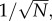 and we repeat the calculation eight times (for an eight processor computer), finding the mean and standard error and then increasing the number of parameter samples if the uncertainty is too great to distinguish between models.

### 4.3. The simulation model

In the simulation model each of *N* = 20 individuals are assumed to be in one of three states: susceptible (*S* is the proportion of individuals in this state); clapping (*I*) and recovered (*R*). Susceptibles have not started clapping yet, while recovered means that the individual has already clapped. The simulation is made in two stages: starting and stopping.

At time *t*(1) = 0, one randomly selected individual starts clapping. We then generate an exponentially distributed random variable, *T*, with rate constant *λ*_2_*S*(*t*)(*I*(*t*) + *R*(*t*)). *T* is the time at which the next individual will start clapping. At *t*(1) = 0, *I*(0) + *R*(0) = 1/*N*, *S*(0) = 1 − 1/*N* and the rate constant is *λ*_2_(1/*N* − 1/*N*^2^). We then set *t*(1) = *t*(0) + *T*, let *I*(*t*) + *R*(*t*) = 2/*N*, *S*(*t*) = 1 − 2/*N* and generate a new exponentially distributed random variable, *T*. This process continues until *S*(*t*(*N*)) = 0. The values *t*(*i*) give the times of the first clap of each of the individuals, *i* = 1 *, …, N*.

To calculate stopping clapping we start at time *t*(1) when the first individual clapped. This individual has performed one clap and will stop clapping with probability
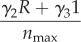
or wait a time *T* ∼ *N*(0.28, 0.09) seconds until the next clap. We update *t*(1) = *t*(1) + *T* to be the time of the next clap by individual 1 and set claps *n*(1) = 2. For all subsequent simulation steps, we find the individual i with the next clap, i.e. *i* for which *t*(*i*) is minimized. Individual *i* will then stop clapping with probability
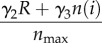
or continue to clap again at a time *T ∼ N*(0.28, 0.09) seconds. If she continues clapping we update *t*(*i*) = *t*(*i*) + *T* to be the time of the next clap by individual 1 and set claps *n*(*i*) = *n*(*i*) + 1. The simulation stops when all individuals have stopped clapping.

Ignoring the effect of number of claps on the probability of stopping, the above clapping model can be expressed in terms of mean-field differential equations4.4
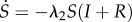
and4.5
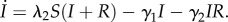
Here, we assume that the per individual rate of starting clapping is proportional to the proportion of those who are either infected or recovered. This is the same as in the simulation model. The main adjustment in the differential equation model is that, since we cannot account directly for the number of claps each individual has done, we instead use the rate *γ*_1_ − *γ*_2_*R* per individual of stopping clapping. The fitted parameters *γ*_1_ = 0.008 per clap and *γ*_2_ = 0.66 per clap are the best model fit when ignoring clap number dependent stopping, i.e. models (1 and 2).

## References

[1] Bikhchandani S Hirshleifer D Welch I 1992 A theory of fads, fashion, custom, and cultural change as informational cascades. J. Polit. Econ. 100, 992–1026 10.1086/261849 (doi:10.1086/261849)

[2] Salganik M 2006 Experimental study of inequality and unpredictability in an artificial cultural market. Science 311, 854–856 10.1126/science.1121066 (doi:10.1126/science.1121066) 16469928

[3] Hedstrom P Liu K-Y Nordvik MK 2008 Interaction domains and suicide: a population-based panel study of suicides in Stockholm, 1991–1999. Social Forces 87, 713–740 10.1353/sof.0.0130 (doi:10.1353/sof.0.0130)

[4] Suchecki K Eguíluz V San Miguel M 2005 Voter model dynamics in complex networks: role of dimensionality, disorder, and degree distribution. Phys. Rev. E 72, 036132 10.1103/PhysRevE.72.036132 (doi:10.1103/PhysRevE.72.036132) 16241540

[5] Castellano C Fortunato S Loreto V 2009 Statistical physics of social dynamics. Rev. Mod. Phys. 81, 591–646 10.1103/RevModPhys.81.591 (doi:10.1103/RevModPhys.81.591)

[6] Ugander J Backstrom L Marlow C Kleinberg J 2012 Structural diversity in social contagion. Proc. Natl Acad. Sci. USA 109, 5962–5966 10.1073/pnas.1116502109 (doi:10.1073/pnas.1116502109) 22474360 PMC3341012

[7] Gallup A Hale JJ Sumpter DJT Garnier S Kacelnik A Krebs JR Couzin ID 2012 Visual attention and the acquisition of information in human crowds. Proc. Natl Acad. Sci. USA 109, 7245–7250 10.1073/pnas.1116141109 (doi:10.1073/pnas.1116141109) 22529369 PMC3358867

[8] Hedstrom P 2005 Dissecting the social: on the principles of analytical sociology. Cambridge, UK: Cambridge University Press

[9] Eagle N Pentland A 2006 Reality mining: sensing complex social systems. Personal and Ubiquitous Computing 10, 255–268 10.1007/s00779-005-0046-3 (doi:10.1007/s00779-005-0046-3)

[10] Song C Qu Z Blumm N Barabasi A-L 2010 Limits of predictability in human mobility. Science 327, 1018–1021 10.1126/science.1177170 (doi:10.1126/science.1177170) 20167789

[11] Néda Z Ravasz E Vicsek T Brechet Y Barabási A 2000 Physics of the rhythmic applause. Phys. Rev. E 61, 6987–6992 10.1103/PhysRevE.61.6987 (doi:10.1103/PhysRevE.61.6987) 11088392

[12] Néda Z Ravasz E Brechet Y Vicsek T Barabási A-L 2000 Self-organizing processes: the sound of many hands clapping. Nature 403, 849–850 10.1038/35002660 (doi:10.1038/35002660) 10706271

[13] Penny W Stephan KE Daunizeau J Rosa MJ Friston KJ Schofield TM Leff AP Kording KP 2010 Comparing families of dynamic causal models. PLOS Comput. Biol. 6, e1000709 10.1371/journal.pcbi.1000709 (doi:10.1371/journal.pcbi.1000709) 20300649 PMC2837394

[14] Mann RP 2011 Bayesian inference for identifying interaction rules in moving animal groups. PLoS ONE 6, e22827 10.1371/journal.pone.0022827 (doi:10.1371/journal.pone.0022827) 21829657 PMC3150372

[15] Mann RP Perna A Strömbom D Garnett R Herbert-Read JE Sumpter DJT Ward AJW 2013 Multi-scale inference of interaction rules in animal groups using Bayesian model selection. PLOS Comput. Biol. 9, e1002961 10.1371/journal.pcbi.1002961 (doi:10.1371/journal.pcbi.1002961) 23555206 PMC3605063

[16] Gautrais J Ginelli F Fournier R Blanco S Soria M Chaté H Theraulaz G Levin SA 2012 Deciphering interactions in moving animal groups. PLOS Comput. Biol. 8, e1002678 10.1371/journal.pcbi.1002678 (doi:10.1371/journal.pcbi.1002678) 23028277 PMC3441504

[17] Sumpter DJT Mann RP Perna A 2012 The modelling cycle in collective animal behaviour. Interface Focus 2, 764–773 10.1098/rsfs.2012.0031 (doi:10.1098/rsfs.2012.0031) 23173077 PMC3499127

[18] Lorenz J Rauhut H Schweitzer F Helbing D 2011 How social influence can undermine the wisdom of crowd effect. Proc. Natl Acad. Sci. USA 108, 9020–9025 10.1073/pnas.1008636108 (doi:10.1073/pnas.1008636108) 21576485 PMC3107299

[19] Katz Y Tunstrom K Ioannou CC Huepe C Couzin ID 2011 Inferring the structure and dynamics of interactions in schooling fish. Proc. Natl Acad. Sci. USA 108, 18 720–18 725 10.1073/pnas.1107583108 (doi:10.1073/pnas.1107583108) 21795604 PMC3219116

[20] Herbert-Read JE 2011 Inferring the rules of interaction of shoaling fish. Proc. Natl Acad. Sci. USA 108, 18 726–18 731 10.1073/pnas.1109355108 (doi:10.1073/pnas.1109355108) 22065759 PMC3219133

[21] Kermack W McKendrick A 1991 Contributions to the mathematical-theory of epidemics 0.1. (reprinted from *Proc. R. Soc. A* **115**, 700–721, 1927). Bull. Math. Biol. 53, 33–55 2059741 10.1007/BF02464423

[22] Granovetter M 1978 Threshold models of collective behavior. Am. J. Sociol. 83, 1420–1443 10.1086/226707 (doi:10.1086/226707)

[23] Sumpter DJT Pratt SC 2009 Quorum responses and consensus decision making. Phil. Trans. R. Soc. B 364, 743–753 10.1098/rstb.2008.0204 (doi:10.1098/rstb.2008.0204) 19073480 PMC2689713

[24] Meunier H Leca J-B Deneubourg J-L Petit O 2006 Group movement decisions in capuchin monkeys: the utility of an experimental study and a mathematical model to explore the relationship between individual and collective behaviours. Behaviour 143, 1511–1527 10.1163/156853906779366982 (doi:10.1163/156853906779366982)

[25] Daley D Kendall DJ 1965 Stochastic rumours. J. Inst. Math. Appl. 1, 42–55 10.1093/imamat/1.1.42 (doi:10.1093/imamat/1.1.42)

[26] Hedström P Bearman P 2011 The Oxford handbook of analytical sociology. Oxford, UK: Oxford University Press

[27] Manski CF 2000 Economic analysis of social interactions, National Bureau of Economic Research Working Paper 7580

[28] Van den Bulte C Lilien GL 2001 Medical innovation revisited: social contagion versus marketing effort. Am. J. Sociol. 106, 1409–1435 10.1086/320819 (doi:10.1086/320819)

[29] Goldstone RL Gureckis TM 2009 Collective Behavior. Top. Cogn. Sci. 1, 412–438 10.1111/j.1756-8765.2009.01038.x (doi:10.1111/j.1756-8765.2009.01038.x) 25164995

[30] Schweitzer F Mach R 2008 The epidemics of donations: logistic growth and power-laws. PLoS ONE 3, e1458 10.1371/journal.pone.0001458 (doi:10.1371/journal.pone.0001458) 18213367 PMC2190793

[31] Jaynes ET 2003 Probability theory: the logic of science. Cambridge, UK: Cambridge University Press

[32] Mäs M Flache A Helbing D Bergstrom CT 2010 Individualization as driving force of clustering phenomena in humans. PLOS Comput. Biol. 6, e1000959 10.1371/journal.pcbi.1000959 (doi:10.1371/journal.pcbi.1000959) 20975937 PMC2958804

[33] MacKay D 2003 Information theory, inference, and learning algorithms. Cambridge, UK: Cambridge University Press

